# Association of Patient and Visit Characteristics With Rate and Timing of Urologic Procedures for Patients Discharged From the Emergency Department With Renal Colic

**DOI:** 10.1001/jamanetworkopen.2019.16454

**Published:** 2019-12-02

**Authors:** Elizabeth M. Schoenfeld, Meng-Shiou Shieh, Penelope S. Pekow, Charles D. Scales, James M. Munger, Peter K. Lindenauer

**Affiliations:** 1 Department of Emergency Medicine, University of Massachusetts Medical School–Baystate, Springfield; 2 Institute for Healthcare Delivery and Population Science, University of Massachusetts Medical School–Baystate, Springfield; 3 School of Public Health and Health Sciences, University of Massachusetts, Amherst; 4 Duke Clinical Research Institute, Division of Urologic Surgery, Duke University School of Medicine, Durham, North Carolina; 5 Department of Medicine, University of Massachusetts Medical School–Baystate, Springfield; 6 Department of Quantitative Health Sciences, University of Massachusetts Medical School, Worcester

## Abstract

**Importance:**

Little is known about the timing of urologic interventions in patients with renal colic discharged from the emergency department. Understanding patients’ likelihood of a subsequent urologic intervention could inform decision-making in this population.

**Objectives:**

To examine the rate and timing of urologic procedures performed after an emergency department visit for renal colic and the factors associated with receipt of an intervention.

**Design, Setting, and Participants:**

This retrospective cohort study used the Massachusetts All Payers Claims Database to identify patients 18 to 64 years of age who were seen in a Massachusetts emergency department for renal colic from January 1, 2011, to October 31, 2014, Patients were identified via *International Classification of Diseases, Ninth Revision* codes, and all medical care was linked, enabling identification of subsequent health care use. Data analysis was performed from January 1, 2017, to December 31, 2018.

**Main Outcomes and Measures:**

The main outcome was receipt of urologic procedure within 60 days. Secondary outcomes included rates of return emergency department visit and urologic and primary care follow-up.

**Results:**

A total of 66 218 unique index visits by 55 314 patients (mean [SD] age, 42.6 [12.4] years; 33 590 [50.7%] female; 25 411 [38.4%] Medicaid insured) were included in the study. A total of 5851 patients (8.8%) had visits resulting in admission at the index encounter, and 1774 (2.7%) had visits resulting in a urologic procedure during that admission. Of the 60 367 patient visits resulting in discharge from the emergency department, 3018 (5.0%) led to a urologic procedure within 7 days, 4407 (7.3%) within 14 days, 5916 (9.8%) within 28 days, and 7667 (12.7%) within 60 days. A total of 3226 visits (5.3%) led to a subsequent emergency department visit within 7 days and 6792 (11.3%) within 60 days. For the entire cohort (admitted and discharged patients), 39 189 (59.2%) had contact with a urologist or primary care practitioner within 60 days. Having Medicaid-only insurance was associated with lower rates of urologic procedures (odds ratio, 0.70; 95% CI, 0.66-0.74) and urologic follow-up (5.6% vs 8.8%; *P* < .001) and higher rates of primary care follow-up (59.2% vs 47.2%; *P* < .001) compared with patients with all other insurance types.

**Conclusions and Relevance:**

In this cohort study, most adult patients younger than 65 years who were discharged from the emergency department with a diagnosis of renal colic did not undergo a procedure or see a urologist within 60 days. This finding has implications for both the emergency department and outpatient treatment of these patients.

## Key Points

**Question:**

When an adult patient is discharged from the emergency department after an episode of renal colic, what is their likelihood of having a urologic procedure within the next 60 days?

**Findings:**

In this cohort study of 66 218 unique index visits by 55 314 patients 18 to 64 years of age, 5.0% of patients underwent a urologic procedure by 7 days and 12.7% underwent a urologic procedure by 60 days. Patients with Medicaid were less likely to undergo urologic procedures.

**Meaning:**

These findings may inform emergency department–based shared decision-making about imaging options and outpatient follow-up for patients younger than 65 years.

## Teaser

This cohort study assesses the rate and timing of urologic procedures performed after an emergency department visit for renal colic and the factors associated with receipt of an intervention within 60 days.

## Introduction

Renal colic results in 1 million to 2 million emergency department (ED) visits per year in the United States and costs patients and insurers more than $10 billion annually.^1,2^ Most patients with renal colic are discharged home from the ED, making it 1 of the 10 most frequent ED discharge diagnoses.^2,3^ Of those top 10, however, the charges for a renal colic ED visit are the highest by 2- to 3-fold, with mean charges from $3500 to $5900, depending on region and payer.^3,4^ These costs are thought to be attributable to the frequent use of computed tomography (CT); an ED visit for renal colic often entails CT and therefore exposure to ionizing radiation because up to 83% of patients undergo CT in the ED.^2^ Although the clinical decision-making involved in ordering a CT often involves prognostication and planning for a possible urologic procedure, the current rate and timing of outpatient procedures are unknown.^5^ Specifically, although several large studies have noted admission procedure rates that vary from 8% to 19% and inpatient procedure rates that vary from 6.3% to 10%,^2,4^ few data exist regarding the intervention rate for patients with renal colic initially discharged from the ED.^5^ Understanding the rate and timing of outpatient interventions could help ED physicians make more judicious decisions about the use of advanced imaging.

Controversy exists within emergency medicine regarding the need for routine CT in patients with suspected renal colic.^6,7,8,9^ Urology guidelines suggest using CT to confirm the diagnosis of stone and to evaluate anatomic characteristics before a stone removal procedure; however, a patient’s likelihood of needing a procedure is generally unknown before the CT, creating a paradox for emergency practitioners.^10^ Patients have also expressed that understanding the risk of radiation exposure and the likelihood of needing a urologic procedure is important to them.^11^ To our knowledge, no population-based studies have characterized the rate and timing of urologic procedures in the cohort of patients who are discharged after an initial visit for renal colic in the United States.^2,4^ Understanding the rate and timing of urologic procedures, as well as the nonclinical factors associated with the performance of a procedure, such as insurance status, could help practitioners and patients make better informed choices regarding the ED diagnosis and management of stones.

In this study, we sought to understand the subsequent health care use among patients seen in the ED for acute flank pain who receive a diagnosis of renal colic. Specifically, we hypothesized that patients discharged from the ED after an index visit for renal colic would have low rates of urologic procedures at 7, 14, 30, and 60 days. We also sought to identify patient- and regional-level factors associated with urologic intervention and to describe practice variation.

## Methods

### Study Design and Setting

We performed a retrospective cohort study using the Massachusetts All Payers’ Claims Database (APCD) from January 1, 2011, to October 31, 2014. This database contains claims from more than 80 payers in Massachusetts, including MassHealth (Medicaid) and all private insurers. Workers’ Compensation, TRICARE and the Veterans Health Administration, the Federal Employees Health Benefit Plan, self-pay, and Medicare are not captured, although claims for patients who are dually enrolled in Medicare and Medicaid are included. The resulting case mix includes most Massachusetts residents younger than 65 years because the state’s uninsured rate (self-pay) is 2.8%.^12^ The data include all claims made, including ED care, inpatient and outpatient care, specialist care, and pharmacy claims and allow for linkages across care, enabling identification of outcomes in subsequent health care interactions. For follow-up care after a procedure, for which payment may be bundled with the procedure, an individual claim may not exist for the follow-up visit. The results of laboratory tests or vital signs are not available in the claims data. The Baystate Medical Center Institutional Review Board reviewed this study, deemed it to be exempt, and granted a waiver of informed consent because deidentified data were used. This study followed the Strengthening the Reporting of Observational Studies in Epidemiology (STROBE) reporting guideline.

The primary aim was to assess the rate and timing of urologic procedures after an ED visit for renal colic. The secondary aim was to identify patient and visit characteristics associated with receipt or timing of a urologic intervention. We hypothesized that despite near-universal health insurance coverage in Massachusetts, Medicaid patients would have lower rates of outpatient urologic interventions. Two concerns motivated this hypothesis. First, Medicaid is often a proxy for poor access to health care services because of lower reimbursement, causing a shortage of practitioners and other access issues, such as transportation-related barriers. Second, patients who are discharged home with renal colic who have a longer wait time before a urologist visit may be more likely to pass their stone and avoid intervention.^13^ For the purposes of informing clinical decision-making, we examined these outcomes in the entire cohort and the subset of patients not initially admitted to the hospital during their index ED visit.

### Selection of Participants

Adult patients aged 18 to 64 years were included if they had an ED visit with an *International Classification of Diseases, Ninth Edition* (*ICD-9*) code consistent with urolithiasis (*ICD-9* codes 788.0, 592.0, 592.1, 592.9, 594.1, 594.2, 594.8, 594.9, and 274.11) from January 1, 2011, to October 31, 2014.^2,14^ The last 60 days of 2014 were omitted to allow for the collection of 60-day follow-up data. When 2 ED visits for an individual met inclusion criteria within 60 days, the first was included as the index visit. In addition, visits were excluded if a urologic procedure occurred within 30 days before the ED visit. If claims were missing an identification number for linkage to other claims or missing the patient’s age or if the patient lived outside Massachusetts, these claims were excluded.

### Measurements

We collected patient characteristics, as available, including age, sex, comorbid conditions, and zip code. Comorbidities were determined via Charlson comorbidity methods.^15^ We decided a priori to include a binary variable for any comorbidities because many young patients with nephrolithiasis do not have documented comorbidities, particularly in claims data for ED encounters. We also included indicators for history of diabetes and renal disease. A binary variable was defined to indicate those with Medicaid only as one group and those with private insurance, other insurance, or multiple payers as another group (all other payers) under the assumption that commercial insurance plans are more comparable to each other than to Medicaid regarding reimbursement and access to care. We collected index visit diagnostic imaging and prescriptions received and filled within 7 days. Data on therapeutics delivered within the ED (such as medications and intravenous fluids) were not consistently recorded and were therefore not included in our analyses. We also categorized dispositions after an initial ED encounter as hospital admission, admission with procedure, or discharge. We collected data on county-level racial/ethnic characteristics, median income, and urologist density (urologists per 100 000 population) for Massachusetts counties from Area Resource Files and assigned patients to counties from their residence zip code or, if missing, to the zip code of the hospital submitting the claim.

Claims with missing data for member-linked identification numbers were excluded because they could not be linked to claims for subsequent health care interactions. Claims with missing ages were also excluded. If a claim for a procedure or outcome (eg, admission) was not found, it was presumed to have not happened and was not considered missing.

### Outcomes

Our primary outcome was an inpatient or outpatient urologic intervention within 60 days after the index ED visit, defined by *Current Procedural Terminology* codes (eTable 3 in the Supplement). We assessed timing of the intervention at 7, 14, 28, and 60 days.^16^ Secondary outcomes included rate of ED return visits within 7 and 60 days (with renal colic–related *ICD-9* codes) and rate of urology and primary care follow-up at 60 days (with any *ICD-9* codes).

### Statistical Analysis

Patient- and visit-level data are presented as number (percentage), mean (SD), or median (interquartile range). Rate and time to urologic procedures are presented as Kaplan-Meier curves for all index ED visits and for patients initially discharged home from the ED. Data were censored at 60 days. Either χ^2^ or 2-tailed *t* tests were used to test for differences in characteristics of patients with and without procedures within 60 days. We used logistic regression to examine factors associated with having a procedure within 60 days among patients initially discharged home from the ED. Cox proportional hazards regression models, adjusted for patient characteristics, were used to test for differences in time to procedure, primary care visit, and urologist visit by insurance status. Because linking claims to specific EDs was not possible, we performed the models again clustering on county of residence (using hierarchical generalized linear modeling) to examine associations with the outcome of intervention (within 60 days of index ED visit). Associations were tested with and without accounting for county-level clustering. *P* < .05 was considered to be statistically significant.

Because patients with a diagnosis of ureterolithiasis who did not undergo CT are often presumed to have ureterolithiasis and therefore could have a lower intervention rate than those with confirmed ureterolithiasis, we performed the time-to-event analysis again with the cohort of patients who underwent CT as part of their ED care. As a sensitivity analysis, we repeated our original analysis, omitting patients with only code 788.0 (renal colic). Analyses were performed with SAS statistical software, version 9.4 (SAS Institute Inc) from January 1, 2017, to December 31, 2018.

## Results

### Characteristics of Visits

**Figure 1.**
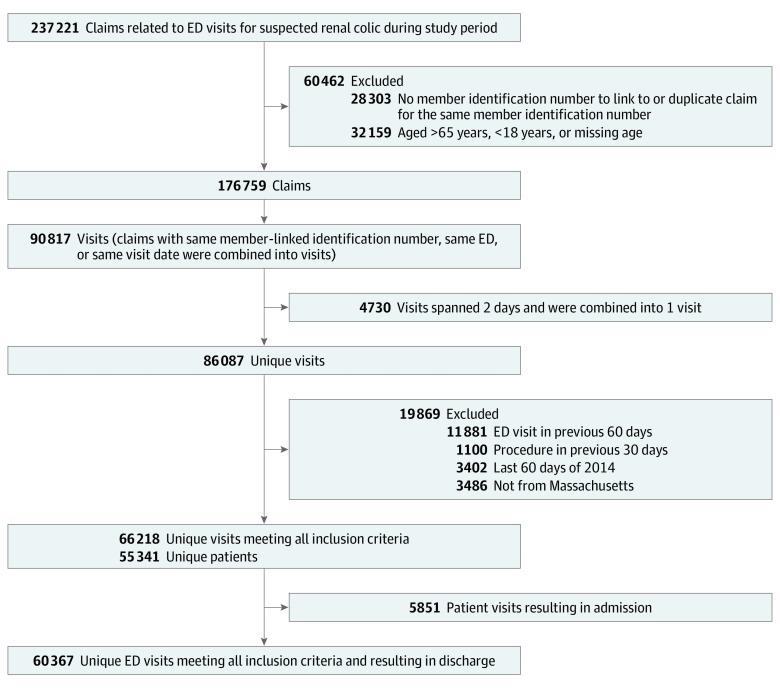
Flow of Included Patients ED indicates emergency department.

Over 200 000 claims were identified, resulting in 66 218 unique visits after exclusions were applied (Figure 1) by 55 314 unique patients (mean [SD] age, 42.6 [12.4] years; 33 590 [50.7%] female; 25 411 [38.4%] Medicaid insured) (Table 1). A total of 41 099 (62.1%) had no documented comorbidities, and 8568 (12.9%) had diabetes. A total of 50 803 (76.7%) underwent CT during their ED visit, whereas 5510 (8.3%) underwent ultrasonography. Of the ultrasonographic examinations, 1245 (22.6%) were coded as limited, consistent with bedside- or emergency physician–performed procedures, whereas 4331 (78.6%) were coded as complete, suggesting radiologist-performed procedures (66 had both). A total of 5851 patients (8.8%) were admitted during the index visit, and 1774 (2.7%) underwent a urologic procedure during their admission. Regarding discharge prescriptions, 15 222 (23.0%) filled a prescription for oral opiates, 7477 (11.3%) for oral nonsteroidal anti-inflammatory drugs, 10 024 (15.1%) for antiemetics, and 11 458 (17.3%) for medical expulsive therapy. The counties represented were 83% white (median) and 11% Latino and had a median household income of $70,386.

**Table 1.** Patient and Visit Characteristics for the Entire Study Cohort

Characteristic	Finding (66 218 Visits and 55 314 Patients)^a^
Age, y	
Mean (SD)	42.6 (12.4)
Median (IQR)	43 (32-53)
Sex	
Female	33 590 (50.7)
Male	32 628 (49.3)
Insurance	
Medicaid only	25 411 (38.4)
Private or combination	40 807 (61.6)
Prescriptions filled within 7 d	
Medical expulsive therapy	11 458 (17.3)
Antiemetic	10 024 (15.1)
Oral opiates	15 222 (23.0)
NSAIDs	7477 (11.3)
ED imaging	
CT	50 803 (76.7)
Ultrasonography	5510 (8.3)
Comorbidity	
Any	25 119 (37.9)
Diabetes	8568 (12.9)
Renal disease	1904 (2.9)
County-level data^b^	
Nonwhite race by county	
Population, %	16.7
Median (IQR)	14.1 (13.0-19.7)
Latino ethnicity	
Population, %	11.4
Median (IQR)	7.8 (7.2-19.2)
Median household income, $	
Mean (SD)	70 386 (14 134)
Median (IQR)	65 735 (59 839-90 025)
Urologists per 100 000 population	
Mean (SD)	4.2 (2.9)
Median (IQR)	3.4 (3.0-5.6)
Outcomes	
Immediate admissions	5851 (8.8)
Immediate admission and procedure	1774 (2.7)
Subsequent ED visit in 7 d	3608 (5.5)
ED visit in 60 d	7654 (11.6)

Abbreviations: CT, computed tomography; ED, emergency department; IQR, interquartile range; NSAIDs, nonsteroidal anti-inflammatory drugs.

^a^ Data are presented as number (percentage) of visits or patients unless otherwise indicated.

^b^ Race and ethnicity data were not available in the primary data set. Patient zip code was linked to county-level data from Area Resource Files. These data represent a county-level proportions, weighted by patients per county.

### Main Results

**Figure 2.**
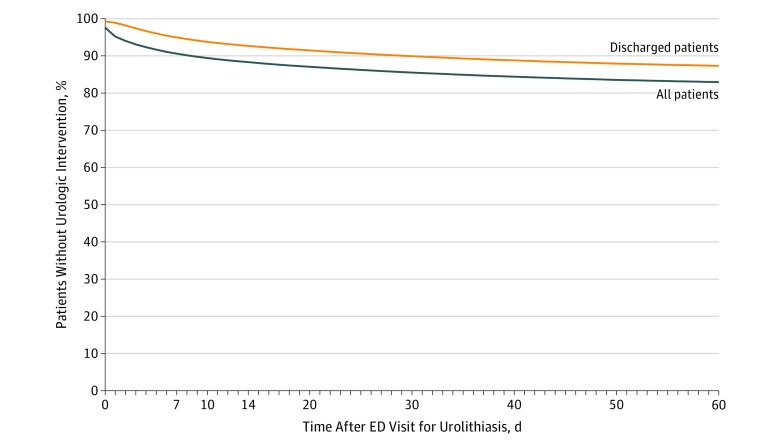
Time From the Index Emergency Department (ED) Visit to Intervention for the Entire Cohort and Those Initially Discharged

The rate and timing of urologic interventions for the entire cohort and the patients initially discharged are shown in Figure 2. For the entire cohort, the rates of urologic intervention were 9.4% at 7 days, 11.7% at 14 days, 14.2% at 28 days, and 17.0% at 60 days. Excluding patients initially admitted to the hospital, the intervention rates were 5.0% (3018 patients) at 7 days, 7.3% (4407) at 14 days, 9.8% (5916) at 28 days, and 12.7% (7667) at 60 days.

Of the 60 367 patients initially discharged from the ED, 52 710 (87.3%) did not undergo a urologic procedure within 60 days, 3226 (5.3%) returned to an ED within 7 days, and 6792 (11.3%) returned to an ED within 60 days. Data on univariate associations of patient factors with procedure within 60 days are given in Table 2.

**Table 2.** Patient and Visit Characteristics for 60 367 Discharged Patients Who Did and Did Not Undergo a Procedure at 60 Days^a^

Characteristic	All Discharged Patients (n = 60 367)	No Procedure Within 60 Days (n = 52 710)	Procedure Within 60 Days (n = 7657)	*P* Value^b^
Age, y^b^				
Mean	42.3	41.9	44.7	<.001
Median (IQR)	43(32-53)	42 (32-52)	46 (35-55)	
Sex				
Female	30 330 (50.2)	26 659 (50.6)	3671 (47.9)	<.001
Male	30 037 (49.8)	26 051 (49.4)	3986 (52.1)	
Insurance				
Medicaid only	23 419 (38.8)	21 118 (40.1)	2301 (30.1)	<.001
Private or combination	36 948 (61.2)	31 592 (59.9)	5356 (70.0)	
Prescriptions filled within 7 d				
Medical expulsive therapy	10 717 (17.8)	8782 (16.7)	1935 (25.3)	<.001
Antiemetic	9506 (15.8)	7926 (15.0)	1580 (20.6)	<.001
Oral opiates	14 200 (23.5)	12 345 (23.4)	1855 (24.2)	.12
NSAIDs	7242 (12.0)	6144 (11.7)	1098 (14.3)	<.001
ED imaging				
Any CT	47 270 (78.3)	41 216 (78.2)	6054 (79.1)	.08
Noncontrast CT	39 126 (64.8)	33 795 (64.1)	5331(69.6)	<.001
Ultrasonography	5168 (8.6)	4672 (8.9)	496 (6.5)	<.001
Comorbidity				
Any	22 291 (36.9)	19 479 (37.0)	2812 (36.7)	.70
Diabetes	7471 (12.4)	6446 (12.2)	1025 (13.4)	.004
Renal disease	1537 (2.6)	1342 (2.6)	195 (2.6)	>.99
County-level data^c^				
Proportion of patients' counties of white race, %				
Mean (SD)	83.3 (7.5)	83.2 (7.6)	84.1 (6.8)	NA
Median (IQR)	86.0 (80.3-87.0)	86.0 (80.3-87.0)	86.1 (80.3-87.0)	
Proportion of patients' counties of nonwhite race				
Mean (SD)	16.7 (7.5)	16.8 (7.6)	15.9 (6.8)	<.001
Median (IQR)	14.1 (13.0-19.7)	14.1 (13.0-19.7)	13.9 (13.0-19.7)	
Proportion of patients' counties of Latino ethnicity				
Mean (SD)	11.0 (7.0)	11.5 (7.2)	11.1 (7.0)	<.001
Median (IQR)	11.5 (7.2-19.2)	11.5 (7.2-19.2)	11.1 (5.4-19.2)	
Median household income, $				
Mean (SD)	70 256 (14 146)	70 256 (14 202)	70 255 (13 749)	.03
Median (IQR)	65 735 (59 839-90 025)	65 735(59 839-90 025)	65 735 (59 839-90 025)	
Urologists per 100 000 population				
Mean (SD)	4.2 (2.9)	4.2 (3.0)	3.9 (2.6)	<.001
Median (IQR)	3.4 (3.0-5.6)	3.4 (3.0-5.6)	3.2 (3.0-4.3)	
Subsequent ED visits				
Subsequent ED visit in 7 d	3226 (5.3)	1981 (3.8)	1245 (16.3)	<.001
ED visit in 60 d	6792 (11.3)	4358 (8.3)	2434 (31.8)	<.001

Abbreviations: ED, emergency department; IQR, interquartile range; NSAIDs, nonsteroidal anti-inflammatory drugs.

^a^ Data are presented as number (percentage) of visits or patients unless otherwise indicated.

^b^ A χ^2^ test was used for association of characteristics with procedure within 60 days except where noted.

^c^ A 2-tailed *t* test was used for difference in means by procedure within 60 days.

**Figure 3.**
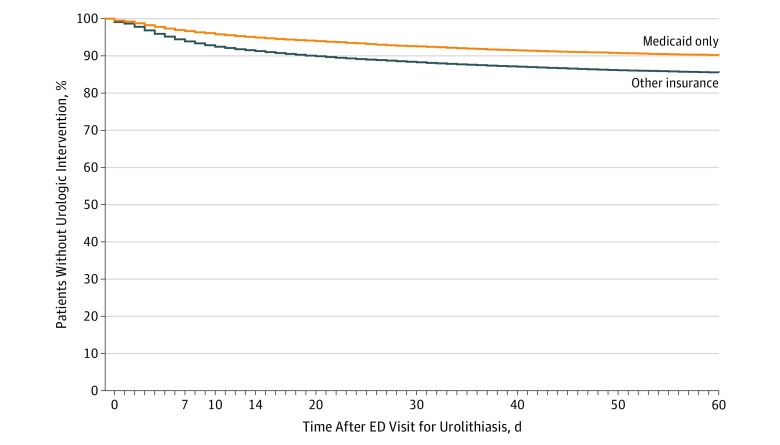
Time From the Index Emergency Department (ED) Visit to Intervention for Discharged Patients With Medicaid-Only Insurance vs All Other Insurance Types

In multivariate analysis, covariates associated with a higher risk of intervention included increasing age and receipt of medical expulsive therapy, prescription nausea medications, and prescription nonsteroidal anti-inflammatory drugs (eTable 1 in the Supplement). Factors associated with a lower risk of intervention included having Medicaid only for insurance (odds ratio, 0.70; 95% CI, 0.66-0.74), undergoing CT (odds ratio, 0.88; 95% CI, 0.83-0.94) or ultrasonography (odds ratio, 0.77; 95% CI, 0.69-0.85) (vs no imaging), and receiving prescription opiates (odds ratio, 0.84; 95% CI, 0.79-0.90). The differences in rate and timing of urologic procedures for Medicaid-only patients vs others is shown in Figure 3. At 28 days, 7.3% of Medicaid patients had undergone a procedure vs 11.4% of patients with other insurance.

These associations did not change when controlling for clustering by county. The density of urologists by county was not associated with receipt of an intervention. The other county-level covariates included were not associated with procedure rates.

In those patients, both admitted initially and discharged, who received a CT in the ED during their index visit, the urologic procedure rate was similar to that of the entire cohort: 8.8% at 7 days, 11.1% at 14 days, 13.7% at 28 days, and 16.5% at 60 days. In the sensitivity analysis, when patients with code 788.0 were omitted, patient characteristics and outcomes did not change substantively (eTable 2 in the Supplement).

### Secondary Analyses

For the entire cohort (admitted and discharged patients), 39 189 (59.2%) had contact with a urologist or primary care practitioner within 60 days (eFigure 1 in the Supplement). At 60 days, Medicaid-only patients had follow-up rates of 5.6% for urology and 59.2% for primary care, whereas those with other insurance coverage had rates of 8.8% for urology and 47.2% for primary care (*P* < .001 for both) (eFigure 2 in the Supplement).

## Discussion

In this large population-based cohort, we linked ED visits with subsequent outpatient care, allowing for a more complete picture of a patient’s health care trajectory after an ED visit for renal colic. In adult patients younger than 65 years who were discharged from the ED with a diagnosis of renal colic, 3226 (5.3%) returned to the ED within 7 days, 3018 (5.0%) had a urologic procedure within 7 days, and 5916 (9.8%) underwent a procedure within 28 days. Of note, the immediate CT rate was 15 times the 7-day procedure rate. Although our findings do not directly assess the clinical impact of CT with subsequent urologic procedures, this marked difference suggests that immediate CT is not routinely necessary to plan for subsequent interventions.

These findings add a key piece of previously missing information that could potentially facilitate shared decision-making. Otherwise healthy patients without signs of infection could undergo ultrasonography as initial imaging because ultrasonography has been recommended by guidelines as the primary diagnostic imaging tool of choice.^10^ If these patients improve clinically and have no indications for immediate admission or procedure, they can be counseled that their risk of needing a urologic procedure in the next 7 days is 1 in 20. With patient input, practitioners could then establish a plan for delayed CT, as needed, based on the patient’s symptoms. A pathway that delays CT has the potential to decrease the radiation burden for this at-risk population, as supported by the current literature^8,17,18,19,20^ and our finding that rates of procedures were similar regardless of whether CT was performed.

Our data allow for a more complete capture of the health trajectory of patients with renal colic than previous studies,^1,2,21,22,23^ many of which have been limited to reporting interventions received during admission at the index visit. In addition, the index admission intervention rate has marked variability; for example, the rate was 7.5% and 52.1% at 2 Canadian hospitals using the same methods.^13^ Variation has also been demonstrated between weekend and weekday admissions.^24^ The cause of this variation is multifactorial,^2,25,26,27^ but it is not clear from our data or from the existing literature why such significant variation in intervention rates exists across studies and regions. Absolute indications for emergency intervention include an obstructing stone in a solitary or transplanted kidney, evidence of systemic infection, and pain or vomiting that fails to improve with initial conservative management.^28^ Relative indications include a stone larger than 1 cm and re-presentation to the ED with renal colic from the same stone.^28^ Current data suggest that operator variability regarding the relative indications does not explain the degree of variation seen, because most stones are smaller than 1 cm and most measures indicating variability have examined index rather than subsequent ED presentation.^2,13,24,29^ There likely is variability in how long practitioners and patients are willing to tolerate conservative management.

Another notable finding from this study is the difference in interventions and urology visits between patients with Medicaid-only insurance and all others. At 28 days, 7.3% of Medicaid patients have undergone a procedure vs 11.4% of patients with other insurance. In our multivariable model, having private or combination insurance coverage (as opposed to Medicaid) had the second strongest association with intervention, after the receipt of a prescription for medical expulsive therapy, which may be associated with presence of a larger stone.^30^ It is unlikely that stone characteristics are different in patients insured by Medicaid, but it is unclear whether the difference in procedure rates represents better or worse care. A recent study^13^ found that earlier intervention for kidney stones was associated with a higher rate of readmissions and subsequent procedures, evidence that increased interventions do not represent improved care. If the Medicaid-only procedure rate represents needed medical care and the rate for insured patients represents overuse, this implies that 4.7% of insured patients received a procedure that they may not have needed.

Similarly, urology office visits rates were lower for Medicaid-only patients (5.6% vs 8.8%). Although this finding may represent patient-related access issues (eg, transportation) or practice-related issues (eg, not accepting Medicaid patients), this has obvious potential to affect patients’ health because nephrolithiasis is a chronically relapsing condition that is somewhat amenable to lifestyle and diet modifications.^1^

Although supplier-induced demand has been blamed for health care use, we did not see any association between urologist density and likelihood of receiving an intervention.^31^ In fact, the mean number of urologists per 100 000 population was higher in the counties with patients who did not have an intervention compared with the counties with patients who had an intervention (Table 2). We were not, however, able to examine the practitioner density at individual hospitals.

### Limitations

This study has limitations. Although this study can tell us what happened after 66 218 ED visits, claims data cannot tell us why.^32^ Claims data represent what was billed, missing many aspects of actual clinical care. Therefore, we were unable to judge the appropriateness of the care provided. In addition, these data are from Massachusetts, and although the data encompass a diverse set of EDs, it is possible that regional variation exists. Previous data have shown that the southern and western regions of the United States have substantially lower admission rates than the Northeast and Midwest (12% in the South and West vs 21% in the Northeast and 19% in the Midwest).^2^ Other regions may have higher initial discharge rates, which may lead to higher outpatient intervention rates. However, if the medical culture is more supportive of conservative management, the intervention rates may actually be lower.

Although we were able to adjust for clustering by county of patient residence, the claims data do not consistently identify individual hospitals; thus, we were unable to assign claims to particular EDs and account for clustering by hospital. It is likely that hospital-level variability is not well captured in these data.^13^ Similarly, the claims data lacked racial and socioeconomic identifiers; thus, county data were used based on zip codes. This method may have obscured associations of race and income with outcomes.

Although these data reveal a lower intervention rate for Medicaid-only patients, they do not offer explanations for this difference. It is possible that this difference is associated with access or different risk factors and needs. It is also unclear whether the difference represents overuse in privately insured individuals or underuse in those with Medicaid or a combination thereof.

In addition, physicians traditionally estimate a patient’s risk of needing an intervention using the size and location of the kidney stone seen on CT.^5^ This study was intended to provide an alternate method of estimation for patients and practitioners that is not based on CT-derived data. However, knowing the stone size and location with use of CT may still provide the most personalized risk assessment. Similarly, older patients may require interventions at different rates than younger patients, and these results should not be extrapolated to patients older than 65 years for whom the benefits of CT may outweigh the risks.

## Conclusions

This is the first study, to our knowledge, to examine population-level post-ED care outcomes for patients with kidney stones. This study adds to the current knowledge about the contemporary diagnosis of kidney stones by demonstrating that in patients younger than 65 years who appeared clinically appropriate for discharge, the intervention rate was potentially low enough to justify a delayed-CT approach. Additional research should focus on risk stratification and shared decision-making tools that incorporate patient-reported outcomes and preferences.

## Notes

Supplement. **eTable 1.** Adjusted Model, Without Clustering by County, for Outcome of Urologic Procedure Within 60 Days of ED Visit (Analysis With Clustering Yielded Similar Results) **eTable 2.** Sensitivity Analysis—Comparison of Complete Cohort to a Cohort That Does Not Include ICD-9 Code 788.0 (“Renal Colic”) **eTable 3.** CPT Codes for Urologic Procedures **eFigure 1.** Claims for Follow-up Care for the Entire Cohort, Within 60 Days **eFigure 2.** Days to Urologic Office Visit (A) and Primary Care Office Visit (B) for Discharged Patients With Medicaid-Only Insurance vs All Other Patients
