# Supplementary material for: Association of Patient and Visit Characteristics With Rate and Timing of Urologic Procedures for Patients Discharged From the Emergency Department With Renal Colic
Source: JAMA Netw Open. 2019 Dec 2;2(12):e1916454. doi: 10.1001/jamanetworkopen.2019.16454 (PMC6902745; doi:10.1001/jamanetworkopen.2019.16454)
Supplement: Supplement. — eTable 1. Adjusted Model, Without Clustering by County, for Outcome of Urologic Procedure Within 60 Days of ED Visit (Analysis With Clustering Yielded Similar Results) eTable 2. Sensitivity Analysis—Comparison of Complete Cohort to a Cohort That Does Not Include ICD-9 Code 788.0 (“Renal Colic”) eTable 3. CPT Codes for Urologic Procedures eFigure 1. Claims for Follow-up Care for the Entire Cohort, Within 60 Days eFigure 2. Days to Urologic Office Visit (A) and Primary Care Office Visit (B) for Discharged Patients With Medicaid-Only Insurance vs All Other Patients [file jamanetwopen-2-e1916454-s001.pdf]

Supplementary Online Content

Schoenfeld EM, Shieh M-S, Pekow PS, Scales CD Jr, Munger JM, Lindenauer PK. Association of patient and visit characteristics with rate and timing of urologic procedures for patients discharged from the emergency department with renal colic. *JAMA Netw Open*. 2019;2(12):e1916454. doi:10.1001/jamanetworkopen.2019.16454

eTable 1. Adjusted Model, Without Clustering by County, for Outcome of Urologic Procedure Within 60 Days of ED Visit (Analysis With Clustering Yielded Similar Results)

eTable 2. Sensitivity Analysis – Comparison of Complete Cohort to a Cohort That Does Not Include ICD-9 Code 788.0 (“Renal Colic”)

eTable 3. CPT Codes for Urologic Procedures

eFigure 1. Claims for Follow-up Care for the Entire Cohort, Within 60 Days

eFigure 2. Days to Urologic Office Visit (A) and Primary Care Office Visit (B) for Discharged Patients With Medicaid-Only Insurance vs All Other Patients

This supplementary material has been provided by the authors to give readers additional information about their work.

© 2019 Schoenfeld EM et al. *JAMA Network Open*.

eTable 1. Adjusted Model, Without Clustering by County, for Outcome of Urologic Procedure Within 60 Days of ED Visit (Analysis With Clustering Yielded Similar Results)

Parameter	Pr > |Z|	Odds Ratio	Odds Ratio Lower Limit	Odds Ratio Upper Limit
Intercept	<.0001			
Sex (Female)	0.1376	1.04	0.99	1.09
Age (10 year intervals)	<.0001	1.17	1.15	1.20
Insurance: Medicaid-only	<.0001	0.70	0.66	0.74
CT Scan Obtained in ED	<.0001	0.88	0.83	0.94
Ultrasound Obtained in ED	<.0001	0.77	0.69	0.85
Medical Expulsive Therapy Prescription Filled	<.0001	1.61	1.51	1.71
Anti-emetic Medications Prescription Filled	<.0001	1.31	1.23	1.40
Oral Opiates Prescription Filled	<.0001	0.84	0.79	0.90
NSAID Prescription Filled	0.0005	1.14	1.05	1.22
With comorbidities	0.12	0.95	0.90	1.01
With Diabetes	0.3685	1.04	0.95	1.13
With Renal Disease	0.8528	1.02	0.86	1.17
Percentage non-white (by county)	0.0055	0.99	0.98	1.00
Percentage Latino (by county)	0.963	1.00	1.00	1.01
Median Household Income per 1000 (by county)	0.1738	1.00	1.00	1.00
Urologists per 100,000 (by county)	0.2129	0.99	0.97	1.01

© 2019 Schoenfeld EM et al. *JAMA Network Open*.

eTable 2. Sensitivity Analysis – Comparison of Complete Cohort to a Cohort That Does Not Include ICD-9 Code 788.0 (“Renal Colic”)

Patient and visit characteristics		All Discharged Patients (including 788.0)	All Discharged Patients (excluding 788.0)
		N (%)	N (%)
		60,367	52766
Age	Mean (median, q1-q3)	42.3 (43, 32-53)	42.6 (43,32-53)
Gender	Female	30,330 (50.2)	26275 (49.8)
	Male	30,037 (49.8)	26491 (50.2)
Insurance	Medicaid Only	23,419 (38.8)	20641 (39.1)
	Private or Combination	36,948 (61.2)	32125 (60.9)
Prescriptions Filled within 7 days	Medical Expulsive Therapy	10,717 (17.8)	9646 (18.3)
	Anti-emetic	9,506 (15.8)	8397 (15.9)
	Oral Opiates	14,200 (23.5)	12563 (23.8)
	NSAIDS	7,242 (12.0)	6423 (12.2)
ED Imaging	Any CT Scan	47,270 (78.3)	43091 (81.7)
	Non-contrast CT	39,126 (64.8)	35372 (67)
	Ultrasound	5,168 (8.6)	4040 (7.7)
Comorbidity	Any Comorbidity	22,291 (36.9)	19745 (37.4)
	Diabetes	7,471 (12.4)	6701 (12.7)
	Renal Disease	1537 (2.6)	1354 (2.6)
County-level data	Non-white % (median, q1-q3)	16.7 (14.1, 13.0-19.7)	16.6 (14.1,13-19.7)
	Latino % (median, q1-q3)	11.5 (7.8, 7.2-19.2)	11.5 (7.8,7.2-19.2)
	Median Household Income	70,256 (65,735, 59,839-90,025)	69874 (65621,59839-90025)
	Urologists per 100,000 mean (median, q1-q3)	4.2 (3.4, 3.0-5.6)	4.1 (3.4,3-5.6)
ED revisits	ED revisit in 7 days	3,226 (5.3)	2849 (5.4)
	ED visit in 60 days	6,792 (11.3)	6013 (11.4)
Procedure within 60 days		7,657 (12.7)	6999 (13.3)

© 2019 Schoenfeld EM et al. *JAMA Network Open*.

eTable 3. CPT Codes for Urologic Procedures

Code	Description
50060	Nephrolithotomy; removal of calculus
50065	Nephrolithotomy; secondary surgical operation for calculus
50070	Nephrolithotomy; complicated by congenital kidney abnormality
50075	Nephrolithotomy; removal of large staghorn calculus filling renal pelvis and calyces (including anatrophic pyelolithotomy)
50080	Percutaneous nephrostolithotomy or pyelostolithotomy, with or without dilation, endoscopy, lithotripsy, stenting, or basket extraction; up to 2 cm
50081	Percutaneous nephrostolithotomy or pyelostolithotomy, with or without dilation, endoscopy, lithotripsy, stenting, or basket extraction; over 2 cm
50130	Pyelotomy; with removal of calculus (pyelolithotomy, pelviolithotomy, including coagulum pyelolithotomy)
50135	Pyelotomy; complicated (eg, secondary operation, congenital kidney abnormality)
50561	Renal endoscopy through established nephrostomy or pyelostomy, with or without irrigation, instillation, or ureteropyelography, exclusive of radiologic
50580	Renal endoscopy through nephrotomy or pyelotomy, with or without irrigation, instillation, or ureteropyelography, exclusive of radiologic service;
50590	Lithotripsy, extracorporeal shock wave
50610	Ureterolithotomy, upper one-third of ureter
50620	Ureterolithotomy, middle one-third of ureter
50630	Ureterolithotomy, lower one-third of ureter
50945	Laparoscopy, surgical; ureterolithotomy
50961	Ureteral endoscopy through established ureterostomy, with or without irrigation, instillation, or ureteropyelography, exclusive of radiologic service;
50980	Ureteral endoscopy through ureterotomy, with or without irrigation, instillation, or ureteropyelography, exclusive of radiologic service; with removal
51060	Transvesical ureterolithotomy
51065	Cystotomy, with calculus basket extraction and/or ultrasonic or electrohydraulic fragmentation of ureteral calculus
52320	Cystourethroscopy (including ureteral catheterization); with removal of ureteral calculus
52325	Cystourethroscopy (including ureteral catheterization); with fragmentation of ureteral calculus (eg, ultrasonic or electro-hydraulic technique)
52330	Cystourethroscopy (including ureteral catheterization); with manipulation, without removal of ureteral calculus
52351	Cystourethroscopy, with ureteroscopy and/or pyeloscopy; diagnostic
52352	Cystourethroscopy, with ureteroscopy and/or pyeloscopy; with removal or manipulation of calculus (ureteral catheterization is included)
52353	Cystourethroscopy, with ureteroscopy and/or pyeloscopy; with lithotripsy (ureteral catheterization is included)

© 2019 Schoenfeld EM et al. *JAMA Network Open*.

eFigure 1. Claims for Follow-up Care for the Entire Cohort, Within 60 Days

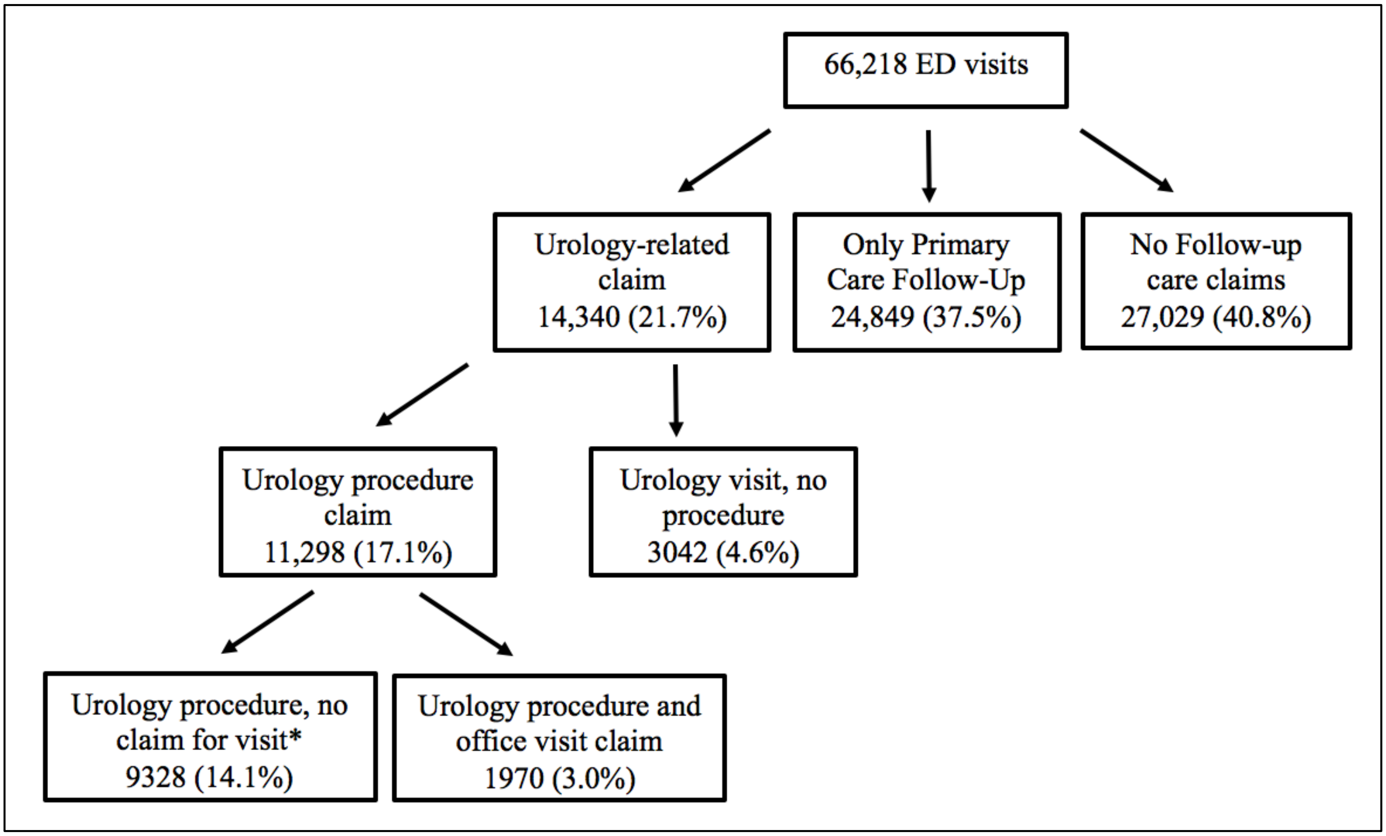

*For patients who received a procedure, follow-up office visits may not incur a separate claim.

© 2019 Schoenfeld EM et al. *JAMA Network Open*.

eFigure 2. Days to Urologic Office Visit (A) and Primary Care Office Visit (B) for Discharged Patients With Medicaid-Only Insurance vs All Other Patients

A.

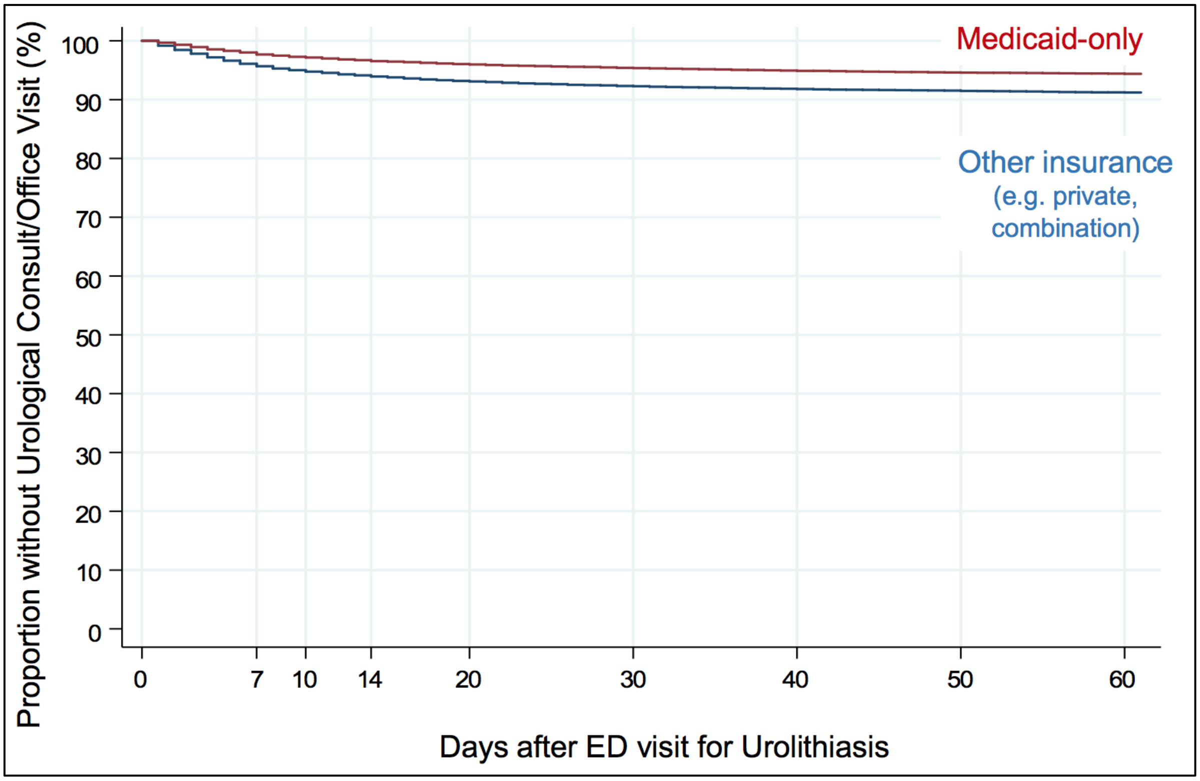

© 2019 Schoenfeld EM et al. *JAMA Network Open*.

B.

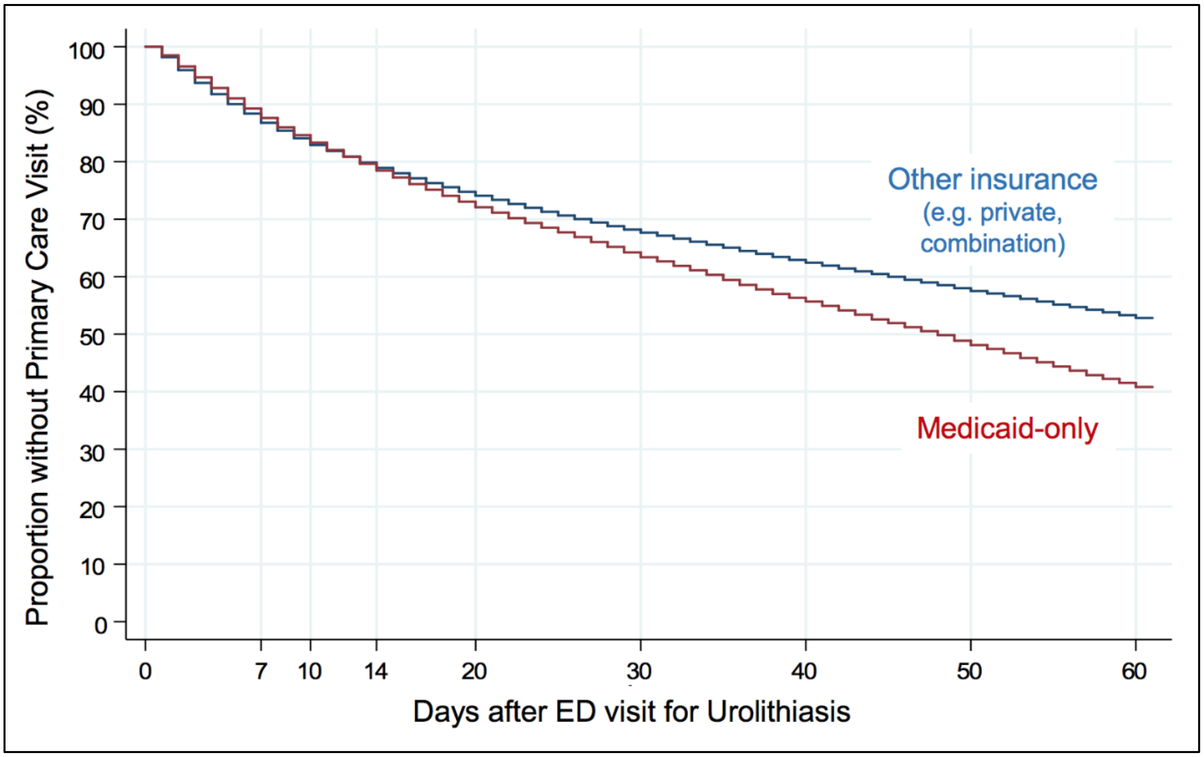

© 2019 Schoenfeld EM et al. *JAMA Network Open*.
